# Acid–base dysregulation and chemosensory mechanisms in panic disorder: a translational update

**DOI:** 10.1038/tp.2015.67

**Published:** 2015-05-26

**Authors:** L L Vollmer, J R Strawn, R Sah

**Affiliations:** 1 Department of Psychiatry and Behavioral Neuroscience, University of Cincinnati, College of Medicine, Cincinnati, OH, USA; 2 Cincinnati Children's Hospital Medical Center, Department of Psychiatry, Cincinnati, OH, USA; 3 Veterens' Affairs Medical Center, Cincinnati, OH, USA

## Abstract

Panic disorder (PD), a complex anxiety disorder characterized by recurrent panic attacks, represents a poorly understood psychiatric condition which is associated with significant morbidity and an increased risk of suicide attempts and completed suicide. Recently however, neuroimaging and panic provocation challenge studies have provided insights into the pathoetiology of panic phenomena and have begun to elucidate potential neural mechanisms that may underlie panic attacks. In this regard, accumulating evidence suggests that acidosis may be a contributing factor in induction of panic. Challenge studies in patients with PD reveal that panic attacks may be reliably provoked by agents that lead to acid–base dysbalance such as CO_2_ inhalation and sodium lactate infusion. Chemosensory mechanisms that translate pH into panic-relevant fear, autonomic, and respiratory responses are therefore of high relevance to the understanding of panic pathophysiology. Herein, we provide a current update on clinical and preclinical studies supporting how acid–base imbalance and diverse chemosensory mechanisms may be associated with PD and discuss future implications of these findings.

## Introduction

Panic disorder (PD) is characterized by spontaneous and recurrent panic attacks that consist of incapacitating periods of acute-onset respiratory, cardiovascular, gastrointestinal, autonomic and cognitive symptoms. PD—which occurs in 6% of Americans^1^—typically begins in the second decade of life^2^ and exhibits a peak prevalence in the third and fourth decades of life.^3^ Thus, this condition is second only to major depressive disorder in terms of associated debility among psychiatric conditions in the United States.^4^ Importantly, PD also represents an independent risk factor for suicidality in diagnostically and demographically heterogeneous clinical populations^5^ and increases the risk of developing other anxiety disorders and secondary mood disorders.^2^ Yet, many patients suffering from PD are not clinically identified and frequently, do not receive even minimally effective treatment.^6^ Even still, available psychopharmacologic treatments (for example, selective serotonin reuptake inhibitors, benzodiazepines) and psychotherapies (for example, cognitive behavioral therapy, prolonged exposure therapy, psychodynamic psychotherapy) or the combination of psychotherapy+pharmacotherapy are often only modestly efficacious (for example, Cohen's *d*=0.4–0.6)^7, 8^ and in some cases (for example, benzodiazepines) may be associated with treatment-specific side effects or risks such as sedation or the risk of dependence or tolerance.

**Figure 1:**
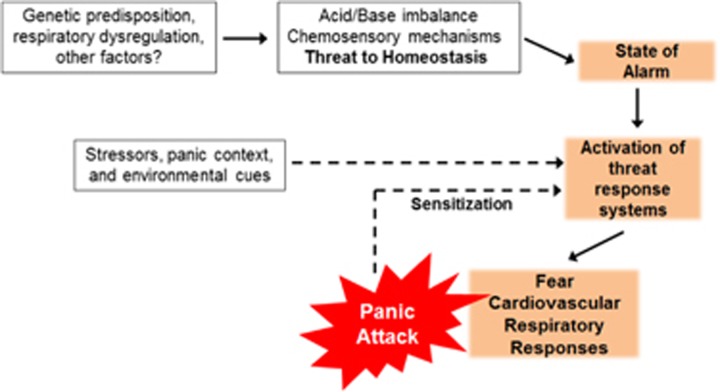
Potential pathogenesis of uncued and cued panic attacks in panic disorder: Initial unexpected attacks may result from an acid/base imbalance or from altered chemosensory mechanisms that represent a ‘threat to homeostasis'. Although the exact origin of pH disturbance is unknown, it may arise due to genetic predisposition, respiratory abnormalities and other factors. This may produce a state of alarm and subsequent activation of threat response systems leading to elevated fear, cardiovascular and respiratory symptoms which, phenomenologically, constitute a panic attack. Further, experiencing uncued panic attacks may sensitize threat response systems to exteroceptive triggers such as stress, panic context and associated phobic cues leading to cued panic attacks. Persistence of uncued and cued panic attacks results in full-blown panic disorder.

Studies elucidating the pathoetiologic mechanisms of PD are urgently needed to reduce morbidity and mortality. Despite the prevalence, as well as associated morbidity and mortality of PD, relatively little regarding the neuropathophysiology of this condition is known. PD is highly heterogenous with variable symptom profile and intensity in panic episodes experienced by the same individual and across patients. According to the DSM-5,^9^ recurrent panic attacks in PD are categorized as being either spontaneous (unexpected) or cued (expected). Collective evidence from challenge studies in the laboratory, neuroimaging, symptomology, treatment responses and translational animal models have led to an increased understanding of PD.^10, 11, 12, 13, 14^ Accumulating evidence suggests that expected panic attacks are triggered by exteroceptive threats (that is, a panic attack context or other unrelated stressors) while spontaneous panic attacks may be provoked by interoceptive sensory triggers caused by fluctuations in the internal homeostatic milieu. An important internal homeostatic trigger for the genesis of panic attacks, supported by an emerging body of work, is acid–base imbalance and associated pH chemosensory mechanisms. Largely founded on panic provocation studies with agents promoting homeostatic pH imbalance and related to the false suffocation alarm theory, the role of acid–base and chemosensory systems in panic provides strong scientific insights on the genesis of uncued panic attacks which may sensitize fear-arousal-stress regulatory circuits to other triggers leading to full-blown PD (Figure 1, cycle of panic). Given the high relevance of interoceptive mechanisms in PD, this review provides an update on our current knowledge and understanding of the role of pH imbalance and chemosensory targets in PD. Although excellent reviews on this topic have appeared previously,^15, 16, 17^ here we focus on (1) current status on pH homeostasis, clinical studies of acid–base physiology and pathophysiology in patients with PD (2) preclinical rodent models of PD, especially those focusing on interoceptive pH imbalance and acid-chemosensory systems recruited in panic-like behaviors, and last (3) synthesize these findings to develop a working neurobiological model of PD that involves dysregulation of central acid sensing and associated circuitry, and finally, (4) translational relevance of these data, gaps in understanding and future implications with a discussion on neuropharmacologic interventions in patients with PD.

## Acidosis, an interoceptive trigger in panic: evidence for relationship between pH disturbances in PD

Many clinical and preclinical studies have focused on dysregulation of central fear circuitry that includes components of the limbic network, which involves connections between the amygdala and the anterior cingulate cortex (Broadman's area 25, 24/32) as well as midbrain regions including the periaqueductal gray matter in panic.^10, 14^ These studies have provided useful information relevant to cued panic attacks and phobias in PD subjects; however, the genesis of unexpected panic attacks still remains elusive to panic researchers. James^18^ first proposed that feelings and emotion can derive from interoceptive sensing of our body states. These internal triggers and interoceptive chemosensory pathways are of particular relevance to PD as initial attacks come ‘out of no-where'. Moreover, accumulating evidence supports a principal role of pH homeostasis in panic physiology and suggests that acidosis may be an interoceptive trigger for panic attacks. Consistent with this, a recent study with ambulatory monitoring, a valid approach for studying spontaneous panic, reported pH disturbances and altered respiratory rhythms in subjects during the final minutes before the onset of a panic attack.^19^ Well-characterized and clinically relevant methods of inducing panic, such as CO_2_ inhalation or sodium lactate infusion, cause acid–base disturbances.^16^ Importantly, CO_2_ inhalation and sodium lactate infusion stimulate respiration, which is itself tightly regulated by pH.^20^ Thus, it is intriguing that despite their disparate effects, respiratory acidosis and metabolic alkalosis, both CO_2_ inhalation and sodium lactate administration lead to extracellular and intracellular acidosis in the brain.^16, 21^ In addition, neuroimaging studies also raise the possibility of dysregulated acid–base buffering and increased plasma and brain lactate responses to metabolic challenges in PD.^22, 23, 24^ There is also a high prevalence of hyperventilation and other respiratory abnormalities among patients with PD.^21, 25, 26^ The link between panic attacks and pH disturbances also forms the basis of the false suffocation alarm theory of spontaneous panic, where CO_2_ hypersensitivity may exist due to a malfunctioning suffocation alarm monitor.^27^ Below we discuss specific areas of investigation that support the panic-pH link.

### Brain pH in patients with PD: evidence from neuroimaging

Neuroimaging studies on PD patients support a role of homeostatic pH disturbances in panic physiology (Table 1). The majority of these studies have focused on lactate responses to homeostatic or activity-dependent challenges. Given the close relationship between lactate and pH in the brain, the findings are consistent with a model of brain metabolic and pH dysregulation associated with altered function of acid-sensitive fear circuits as a trait vulnerability factor in PD. Exaggerated activity-dependent brain lactate responses are observed in PD patients, even remitted patients, as compared with healthy controls, suggestive of underlying pH abnormalities.^23, 28^ Further, activity-dependent changes in glutamate–glutamine were smaller in PD patients.^28^ Others have shown increased (and more prolonged) brain lactate levels compared with healthy comparison subjects during sodium lactate infusion, although resolution was limited.^29^ In a subsequent study, the greater and prolonged brain lactate rises in the insula in patients with PD during and following lactate infusion^30^ were observed—a finding of great importance given the central role of the insula in interoceptive pathways.^31^ Interestingly, differential brain metabolic responses fail to normalize following treatment with the selective serotonin reuptake inhibitor fluoxetine suggesting that abnormal metabolic lactate responses represent a trait feature of PD. Accentuated increases in brain lactate levels have also been reported in PD subjects during hyperventilation, that promotes pH imbalance, as compared with comparison subjects,^22^ suggesting that increased lactate possibly reflects altered cerebral blood flow in response to hypocapnia evoked by increased breathing. Using phosphorous (^31^P) spectroscopy, pH dynamics during hyperventilation were assessed by Friedman *et al.*,^32^ and reported an abnormal pH-buffering capacity in PD. Very recently, CO_2_-evoked activation in the brain stem area was reported in panic patients, controls and divers (representing a group with reduced CO_2_ sensitivity).^33^ The authors reported significant increases in activation within brain stem and insular areas in panic patients as compared with controls and diver groups supporting the role of these areas in interoceptive sensing and processing in PD. Recently, new magnetic resonance imaging techniques such as the T_1_ relaxation in the rotating frame (T_1_ρ) have been developed and have facilitated greater pH sensitivity and improved temporal and spatial resolution over ^31^P spectroscopy.^34^ Increased T_1_ρ was observed in PD patients in the occipital cortex as compared with healthy controls^35^ consistent with abnormal pH regulation in PD. Interestingly, the magnitude of the T_1_ρ response correlates with the severity of anxiety symptoms in patients with PD (but not in healthy subjects). Thus, it is increasingly apparent that this new imaging technique will allow for greater understanding of pH-dependent changes in PD. Finally, although studies of panic symptom provocation are still needed, evidence from functional magnetic resonance imaging and functional magnetic resonance imaging supports differences in metabolic activity or other factors such as cerebral blood flow may lead to altered pH homeostasis in PD.

### Symptom provocation challenge studies with agents producing acid–base imbalance

Panic attacks can be induced in PD individuals by a variety of agents such as CO_2,_^36, 37^ sodium lactate,^38^ doxapram,^39^ isoproterenol,^40^ caffeine,^41^ yohimbine,^42^ cholecystokinin (CCK-4 and agonists),^43^ the benzodiazepine receptor antagonist, flumazenil,^44^ serotonin receptor agonists^45^ and opioid receptor antagonists.^46^ It is important to note, however, that homeostatic perturbations such as CO_2_, lactate and doxapram that cause direct or compensatory pH shifts reliably evoke panic attacks that closely resemble spontaneous panic attacks. Furthermore, as opposed to other challenges, these agents induce panic attacks specifically in PD subjects versus other disorders such as depression, premenstrual dysphoric disorder, generalized anxiety disorder and posttraumatic stress disorder.^47, 48^ Panic provocation agents linked to homeostatic pH imbalance are discussed below.

#### CO_2_ inhalation

Inhalation of CO_2_—a commonly studied interoceptive stimulus—produces intense fear, autonomic and respiratory responses that can evoke panic attacks in individuals with PD. For this reason, CO_2_ is frequently used as a biological challenge and pathological marker of PD.^11, 36^ First described in 1951 by Cohen and White,^37^ CO_2_ inhalation is established as a reliable panicogen in patients with PD.^11, 36, 49, 50^

The partial pressure of CO_2_ in the blood increases following CO_2_ inhalation challenge. In addition, and of direct relevance to central nervous system (CNS) physiology, CO_2_ readily crosses the blood–brain barrier and is sensed by H^+^ and CO_2_ chemoreceptors in the CNS and periphery.^51^ In the extracellular fluid, CO_2_ is hydrolyzed to carbonic acid (H_2_CO_3_) by carbonic anhydrase which readily dissociates into bicarbonate (HCO_3_^-^) and H^+^.^51^ The resulting acidosis is thought to be the trigger for the panic symptoms caused by this challenge including hyperventilation and increased blood pressure.^52^ Klein puts forth in his false suffocation theory that hyperventilation may have a protective role to combat attacks caused by increases in CO_2_ (acidosis).^27^ However, this is a faulty response because the respiratory alkalosis caused by hyperventilation is always associated with a compensatory metabolic acidosis produced by tissue buffering systems that release H^+^ ions.^21^ Panic challenge studies with acetazolamide shed light on the role of protons as effector molecules for generating panic responses; acetazolamide, a carbonic anhydrase inhibitor, blocks the facilitated conversion of CO_2_ to bicarbonate and H^+^, leading to increases in CO_2_ concentrations. Interestingly, administration of intravenous acetazolamide fails to induce panic attacks in patients with PD^53, 54^ suggesting that H^+^ ions, rather than CO_2_ *per se* may facilitate panicogenesis.

Currently, two CO_2_ inhalation techniques are used in panic challenge studies. In the first, steady-state inhalation, a low concentration of CO_2_ (5–7.5%) is inhaled for approximately 1–20 min or until a panic attack occurs. In the second approach, individuals inhale a high concentration of CO_2_ (35%).^36^ The advantage of modeling CO_2_-induced panic is that these CO_2_-induced panic attacks closely resemble spontaneous panic attacks and the attacks resolve quickly.^11^ Interestingly, although PD is twice as likely to occur in women,^3^ sex differences in CO_2_-reactivity are less clear. Although there is some evidence that women report greater fear and anxiety following a CO_2_ challenge,^55, 56, 57^ not all studies have observed gender effects.^50, 58, 59^

CO_2_ inhalation has also been useful for exposure-based treatments in patients with PD^60, 61^ and has been utilized for validation of current treatments such as selective serotonin reuptake inhibitors: paroxetine, sertraline, fluvoxamine^62^ and benzodiazapine alprazolam.^63^ In addition, screening of potential anti-panic medications such as CRF1 receptor antagonist, R317573,^64^ GABA agonist: zolpidem^63^ and neurokinin-1 receptor antagonist: vestipitant^65^ has also been conducted using this challenge. Thus, CO_2_ inhalation appears to have utility for testing the efficacy of pharmacotherapeutic agents and for identifying vulnerability to PD.

#### Sodium lactate infusion

In addition to CO_2_, sodium lactate is a reliable panicogen^38^ frequently used in challenge paradigms. A masked intraveneous infusion of a 0.5 M sodium lactate (10 ml kg^−1^) produces panic attacks in vulnerable individuals.^38, 66^ Lactate-induced panic attacks, like CO_2_-induced panic attacks, phenomenologically mirror spontaneous panic attacks (that is, symptoms of dyspnea, generalized fear, a desire to flee and fear of losing control.^67^ Clinically, susceptibility to lactate-induced panic attacks are frequently used as treatment outcome measures for psychopharmacologic treatments.^68, 69, 70^

A byproduct of cellular metabolism, lactate serves as an energy source for neurons,^71^ and alters systemic acid–base balance. Pertinent to lactate infusions, lactate can cross the blood–brain barrier through monocarboxylate transporters and there is evidence that lactate becomes a significant fuel source in the brain when elevated in blood.^72^ When administered intravenously to lower primates, lactate decreases brain pH^73^ as H^+^ is co-transported with lactate via monocarboxylate transporters. Although lactate infusion may evoke acidosis, a direct role of pH in lactate-evoked panic has not been demonstrated. Interestingly, patients with PD show exaggerated lactic acid production in response to alkalosis evoked by sodium lactate infusion suggestive of increased compensatory drive and impaired acid–base buffering in these individuals.^17^ Other studies reported that a rapid overload of sodium and resultant acute hypernatremia may contribute to sodium lactate-evoked panic since hypertonic saline (3%) facilitated panic symptoms similar to 0.5 M sodium lactate.^74^ An interesting observation in the study was the induction of mild acidosis by hypertonic saline while sodium lactate-evoked hyperventilation and associated alkalosis, although specific parameters such as blood pCO_2_ were not measured. Lactate-evoked panic attacks do not recruit neuroendocrine responses as a dissociation between autonomic activation and cortisol has been reported in ‘panickers' following sodium lactate.^75^ Potential downstream mechanisms for lactate sensitivity in PD are not clear. Involvement of GABAergic system has been suggested by effective blockade of lactate-evoked panic in subjects treated with gabapentin,^76^ while presynaptic, α2adrenergic agonist, clonidine had partial effects. Additionally, concentrations of endogenous neuroactive steroid modulators of the GABA_A_ receptor, allopregnanolone and pregnanolone are decreased in patients with PD during lactate-evoked panic.^77^ Elegant preclinical studies by Shekhar and colleagues have highlighted the role of circumventricular organs (CVOs), hypothalamic GABA, angiotensin and orexin systems in sodium lactate-evoked panic responses (see section ‘Sodium lactate rodent model and hypothalamic GABAergic and acid-chemosensitive orexin targets'). Thus, the underlying mechanism(s) or a direct role of acidosis in sodium lactate-induced panic attacks has not been elucidated to date, and while lactate may contribute to decreased brain pH, the exact effector in lactate-evoked panic in PD subjects is still unclear.

#### Doxapram

Doxapram, a respiratory stimulant first synthesized in 1962 (Ward and Franko^78^) has been examined in the management of acute respiratory failure during the 1960s and 1970s and likely had a specific role in the treatment of individuals with chronic obstructive pulmonary disease. Specifically, administration of doxapram increases tidal volume and ventilation frequency.^79^ Clinically, doxapram use is primarily limited to post-anesthesia shivering prophylaxis and stimulation of respiratory drive in premature infants. However, its use is also associated with second-degree atrioventricular block and QTc prolongation. Regarding patients with PD, doxapram has been examined in several studies, given its tendency to cause hyperventilation. In this challenge, doxapram (0.5 mg kg^−1^, IV)^39, 80, 81^ produces panic attacks that phenomenologically mirror spontaneous panic attacks with associated hyperpnea, tachycardia, increased blood pressure and fear symptoms. Contribution of forebrain regions in doxapram-evoked panic is supported by recent studies where doxapram was administered to PD patients and healthy subjects before positron emission tomography with ^18^F-deoxyglucose.^82^ Cardiac responses were accentuated and patients with PD exhibited decreased prefrontal activity (relative to controls) and increased activity within the cingulate gyrus and amygdala, suggesting a failure to activate prefrontal inhibitory structures in patients with PD.

The underlying mechanism, specifically a role of pH in doxapram-evoked panic has not been established. Hyperventilation induces alkalosis, which has been reported to evoke a compensatory increase in lactic acid release; a response that is exaggerated in PD patients.^17^ It is unclear, however, whether this compensatory increase in acidosis is associated with doxapram-evoked panic attacks. There is evidence that the effects of doxapram may be related to the inhibition of TASK-1 and TASK-3 acid-sensitive potassium channels located in brain stem serotonergic neurons.^83, 84^ In this regard, inhibition of the TASK-1 and TASK-3 channels could increase the excitability of brain stem pH-sensitive neurons and may link the panicogenic action of CO_2_ inhalation and doxapram administration.^85^ In addition, increased respiratory drive by doxapram may aggravate a pre-existing respiratory abnormality in PD. As for lactate, a potential role of pH and acidosis in panic provocation by doxapram may be speculated, however, direct evidence for this link is currently lacking.

### Genetics

A strong contribution of genetics and family history in PD prevalence was first reported by Crowe and colleagues.^86^ In support of a genetic component in vulnerability to interoceptive triggers and PD, higher sensitivity to 35% CO_2_ was observed in first-degree relatives of patients with PD.^87^ CO_2_ hypersensitivity has been proposed as a genetic risk and disease-specific trait marker for PD^88^ also supported by twin studies.^89, 90^ Importantly, a distinction between genetic vulnerability to CO_2_ hypersensitivity versus trait anxiety experienced pre-CO_2_ inhalation was found suggesting that there are specific genetic factors associated with responsivity to stimulation via CO_2_ versus factors related to underlying trait anxiety.^91^

However, as PD does not develop in all individuals with CO_2_ hypersensitivity, it underscores the relevance of other risk factors for development of PD. A combination of genetic factors and early adversity such as childhood parental loss may determine hypersensitivity to CO_2_ and PD.^92^ An interesting preclinical study in cross-fostered mice pups revealed persistent expression of enhanced CO_2_-evoked respiratory responses in mice with a history of interference with dam–pup interactions suggestive of significant gene-by-environment effects on heightened CO_2_ sensitivity.^93^ In any case, hypersensitivity to elevated CO_2_ may help identify childhood groups at familial risk for subsequent development of PD^94, 95^ Interestingly, association of polymorphisms within the tryptophan hydroxylase-2 (TPH-2) gene and CO_2_ responses is observed suggestive of a role of the serotonergic (5-HT) system in the effects of CO_2_.^96^ Accumulating evidence strongly supports an association of polymorphisms in multiple markers of the 5-HT system, including polymorphisms in the gene locus and 3' polyadenylation site of the serotonin transporter (5-HTT) with PD.^97, 98^ An association of 5-HT biosynthetic enzyme TPH-2 and 5-HT receptor subtypes R1 and R2 with PD has also been reported.^99, 100, 101^ The 5-HT system is of interest given its role in the regulation of panic-like behaviors.^102^ Importantly, evidence of chemosensory serotonergic neurons in the medullary raphe (see section ‘Acid chemosensory serotonergic neurons in the medullary raphe nucleus') underscores the role of the 5-HT system in translation of pH fluctuations to panic-relevant ventilatory responses.

Lactate sensitivity on the other hand, did not show familial vulnerability.^103^ However, an association of polymorphism within the exon of the lactate dehydrogenase A gene was reported with CO_2_ sensitivity where the LDH polymorphism was a risk factor for increased CO_2_ responses.^104^ This is relevant, given the role LDH in lactate metabolism and its dependence on cell pH.

A recent study identified two single nucleotide polymorphisms within the acid sensing ion channel 1 (ASIC1) gene, ACCN2 in individuals with PD, which was associated with increased amygdala volume and hyperactivity in these subjects.^105^ This observation strongly supports an association of altered pH sensing within the amygdala with increased risk for PD. Collectively, all evidence support a strong genetic vulnerability component to interoceptive threats (represented by CO_2_ inhalation) and polymorphisms in chemosensory targets such as ASICs. However, other environmental factors may be required for the development of symptomology of PD.

Collectively, evidence from neuroimaging, challenge studies for panic provocation and genetics support that pH homeostasis and acid–base disturbances may contribute to PD at least in a large subset of patients with PD. In the sections below, we discuss pH chemosensory mechanisms and our understanding so far on their potential contributions to panic pathophysiology using preclinical models.

## pH chemosensory molecules and their relevance to panic pathophysiology: evidence from preclinical studies

**Figure 2:**
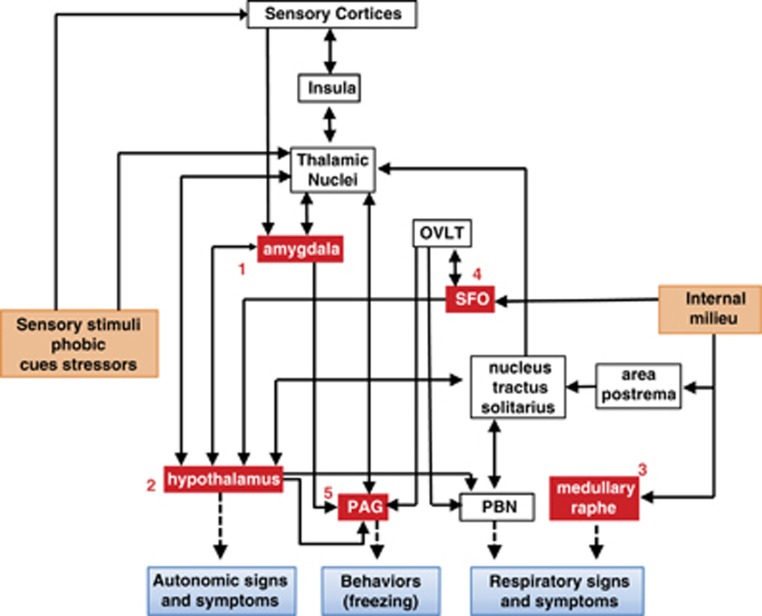
Localization of chemosensory targets and regional circuits contributing to genesis and expression of panic. 1: acid sensing ion channels (ASICs) in the amygdala, 2: orexin neurons in the hypothalamus, 3: serotonergic neurons in the medullary raphe, 4: T-cell death-associated gene-8 receptor in the subfornical organ (SFO), 5: hypoxia-sensitive chemosensory neurons in the periaqueductal gray (PAG). Regions such as the SFO and medullary raphe can directly detect pH fluctuations in the internal milieu, while the hypothalamus, amygdala and PAG in addition to their chemosensory potential also represent key nodes in the processing of external threats, and sensory stimuli. Uncued panic may arise due to homeostatic imbalance in pH in the brain and internal milieu. Acidosis ‘sensed' by chemosensory mechanisms may be translated to autonomic, behavioral and respiratory symptoms of a panic attack. The amygdala, PAG and the hypothalamus can regulate behavioral and autonomic symptoms of panic, whereas respiratory symptoms may be regulated by brain stem regions such as the medullary raphe and the parabrachial nucleus (PBN) via inputs from the hypothalamus and indirectly from the SFO through the organum vasculosum of the lamina terminalis (OVLT). Many of these structures via thalamic nuclei connect with the insula, a region relevant for interoceptive sensing and shown to be dysfunctional in PD. Cued panic attacks may be an outcome of sensory stimuli and phobic cues associated with previous attacks or stressors relayed via sensory cortices and thalamic nuclei to the amygdala and the hypothalamus. It is important to note the overlap and connectivity between pH chemosensory regions and exteroceptive threat processing areas suggesting that uncued and cued panic may recruit similar underlying circuitry depending on modality of the trigger leading to panic.

Acid chemosensory mechanisms become relevant since they can detect and translate interoceptive pH imbalance that may exist in PD (see above). Given the intense respiratory symptoms experienced during a panic attack, it is logical to conclude that these sensing mechanisms are recruited in panic, since respiration is tightly regulated by pH. Multiple chemosensitive areas within the caudal brain stem such as the retrotrapezoid nucleus, solitary nucleus, locus coeruleus and the medullary raphe nuclei elicit neuronal responses to hypercapnia and regulate respiration.^51, 106^ Although chemosensory mechanisms for pH are generally regarded as a brain stem phenomenon, studies in the past decade have revealed several non-brain stem regions to participate in pH chemosensation and associated behavioral and physiological responses. Primary among these are the amygdala, dorsomedial/perifornical hypothalamus and the periaqueductal gray, although other structures such as CVOs may also participate as chemosensitive sites.^107, 108^ In fact, the concerted activity as well as, anatomical and functional links between rostral forebrain and brain stem structures is pertinent to panic attacks, where intense respiratory and psychological symptoms coexist. Figure 2 shows the localization of PD-relevant pH chemosensory targets, based on evidence derived from preclinical studies, and associated circuits that might contribute to panic responses. It is important to note that these regions closely overlap with circuits regulating emotional/behavioral responses, autonomic function and respiration. In addition to regulation of fear and anxiety, these areas send projections to caudal brain stem areas thereby impacting respiratory outcomes. The following section describes specific chemosensory targets as well as panic-relevant preclinical paradigms that support their contributions to PD. Although no animal model can simulate all aspects of panic pathophysiology, the unique feature of panic provocation with acid–base modulators has enabled simulation of human panic in preclinical setting.

### Acid-sensing ion channel

The most well-characterized acid-chemosensory target in terms of PD relevance is the acid-sensing ion channel 1 (ASIC-1a), a voltage-insensitive H^+^-gated cation channel located on neurons in the CNS (reviewed in Wemmie^15^). ASIC-1a has high levels of expression in the amygdala, dentate gyrus of the hippocampus, cortex, striatum and nucleus accumbens,^109, 110^ regions that are components of the limbic–corticostriatal loop, which is thought to be involved in assigning emotional valance to external stimuli.^109^ The contribution of ASIC-1a localized in the amygdala has been studied using translational as well as clinical studies using CO_2_ inhalation. Mice lacking ASIC-1a show decreased fear responses to CO_2_ inhalation.^111^ As shown in that study, CO_2_ inhalation reduced amygdalar pH to an extent that activates ASIC-1a.^111^ Furthermore, only control mice, not ASIC-1a knockout mice, freeze in response to lowered amygdala pH (secondary to injection of acidified artificial cerebrospinal fluid). In addition, restoration of ASIC-1a expression in the amygdala of knockout mice is associated with a return of freezing responses to CO_2_. Collectively, these results suggest that the amygdala is an acid-chemosensitive site and that ASIC-1a within the amygdala mediate CO_2_-evoked fear responses. However, ASIC-1a knockout mice also demonstrate attenuated fear responses during context and cued fear conditioning^109^ and exposure to predator odor challenge,^112^ suggesting that that the regulation of fear by ASIC-1a extends to exteroceptive stimuli and raising the possibility that ASIC-1a may not be selective to interoceptive threats. Genetic studies in humans have linked ASIC polymorphisms to amygdala volume and activity^105^ suggesting contributions of this chemosensor to amygdala function. In addition, recent studies in humans with bilateral amygdala damage resulting from a rare autosomal recessive disorder, Urbach–Wiethe disease in which there is bilateral destruction of the amygdala, have questioned the necessity of the amygdala in panic responses to interoceptive threats such as CO_2._^113^ As reported in their study, patients with neurodegenerative damage in the amygdala exhibited panic responses following CO_2_ inhalation challenge. Further, physiological responses to CO_2_ inhalation such as, ventilation and heart rate, were increased as compared with non-panicking controls and similar to the PD patients. In previous studies, fear responses to exteroceptive threats, such as visiting a haunted house and watching video clips of scary movies were blunted in these patients.^114^ All together, their results suggest that acid-chemosensory mechanisms in other brain regions may orchestrate fear and panic responses to interoceptive stimuli.

### Sodium lactate rodent model and hypothalamic GABAergic and acid-chemosensitive orexin targets

The rodent sodium lactate model of panic pioneered by Shekhar and colleagues provides an excellent translational paradigm in terms of face, predictive and construct validity for sodium lactate challenge in humans (for review see Johnson and Shekhar^115^). Interestingly, infusing lactate alone does not elicit panic-like behavior in rodents, rather, chronic inhibition of GABAergic tone in the dorsal medial-perifornical hypothalamus is necessary for the expression of lactate-evoked responses. In this regard, rats with tonic inhibition of hypothalamic GABA exhibit intense behavioral, respiratory and cardiovascular responses following sodium lactate infusion.^115, 116^ Increased anxiety-like behaviors in the social interaction test and elevated-plus maze tests, elevated blood pressure, tachycardia and hyperventilation was observed in GABA-compromised, lactate-infused rats and these behavioral and physiological responses mirror those observed in PD patients challenged with sodium lactate.^38^ Electrical stimulation of the ventromedial hypothalamus in humans leads to tachycardia and panic (self-reported).^117^ Along with simulation of panic symptoms, this model is responsive to anti-panic and anxiolytic medications, including the benzodiazepine alprazolam. This model also highlights an important role of sensory CVOs as upstream sites for detection of interoceptive stimuli such as sodium lactate.^118^ Further investigation revealed that sodium (but not lactate or osmolality) is the primary trigger that is sensed via sodium ion channels in the anterior third ventricle region. Involvement of hypothalamic angiotensin II and orexin in orchestrating panic-like responses to lactate was confirmed using selective antagonists and RNAi probes.^119, 120^ Orexin expressing neurons in the hypothalamus are of particular interest to panic physiology. In addition to being essential to the effects of sodium lactate, these have been identified as chemosensitive.^121, 122^ Blunted respiratory responses to hypercapnia were seen in mice lacking prepro-orexin.^123^ Antagonism of the ORX1 receptor with SB334867 attenuates CO_2_-induced respiratory responses in mice,^123^ whereas anxiety and hypertensive responses to CO_2_ inhalation require activation of the Orexin-1 receptor.^124^

Thus, converging evidence from the lactate and CO_2_ inhalation model suggests that orexin antagonists may be an attractive therapeutic option for PD, although prolonged suppression of the orexin system may have its own complications.^125^

### Acid chemosensory serotonergic neurons in the medullary raphe nucleus

The serotonergic raphe neurons in the brain stem detect decreases in pH due to hypercapnia^126, 127^ and are strategically located near large arteries where they are able to sense levels of CO_2_ in the blood and quickly initiate behavioral and autonomic responses to maintain homeostasis.^128^ These serotonergic neurons may be involved in the panic-like responses of CO_2_ due to their projections to the anterior limbic and prefrontal fear-processing circuits.^126^ In addition, silencing these pH-sensitive serotonergic neurons in lower animals disrupts chemosensitive responses to CO_2_ inhalation that impair respiration.^129^ Recent studies using un-anesthetized *in situ* perfused decerebrate brain stem preparations suggest that the medullary raphe also contains non-serotonergic, CO_2_-chemosensitive neurons that are positive for neurokinin-1 receptor, suggesting other targets for CO_2_-evoked ventilatory responses.^130^ An interesting study reported significantly lower distress and breathlessness to CO_2_ inhalation in subjects with acute tryptophan depletion suggesting role of serotonin in promoting aversive respiratory sensations to hypercapnia.^131^

Taken together, these data suggest a potential relevance for brain stem acid-chemosensory neurons in respiratory distress, of relevance to PD. However, additional studies especially panic-relevant models need to be explored.

### Acid-sensing T-cell death-associated gene-8 receptor

The T-cell death-associated gene-8 (TDAG8) receptor, an acid-sensing G-protein-coupled receptor (GPCR) located on immune cells in the CNS and periphery,^132, 133, 134^ was originally identified by its increased mRNA expression during programmed cell death of mouse thymocytes mediated by T-cell receptor engagement,^132^ but is increasingly recognized for its putative role in the pathoetiology of panic-like behaviors in lower animals. TDAG8 is a member of the ‘G2A' group of GPCRs, which includes the G2 accumulation receptor (G2A), ovarian cancer G-protein receptor 1 (OGR-1), and G-protein-coupled receptor 4 (GPR4).^132^ Although these receptors were originally characterized as lysolipid receptors, they were later found to sense extracellular protons resulting in the stimulation of intracellular signaling pathways.^134, 135, 136, 137, 138^ Accumulation of cyclic adenosine 5'-monophosphate (cAMP) has been observed in cells transfected with mouse and human TDAG8 cDNA to low extracellular pH.^138^ To date, TDAG8 is the only proton-sensing GPCR expressed in brain tissue. Recent studies by our group has characterized TDAG8 expression in the CNS, and found it to be enriched in sensory CVOs, which include the subfornical organ, organ vasculosum of the lamina terminalis and the area postrema.^108^ The sensory CVOs are specialized chemosensory regions that are highly vascularized and lack the blood–brain barrier.^139^ The sensory CVOs contain cellular contacts with the blood and the cerebrospinal fluid allowing them to relay signals from blood and cerebrospinal fluid to autonomic control centers of the brain.^140^ Sensory CVOs have been linked to panic via their ability to sense panic-stimuli in the circulation and activate downstream targets via their efferent and afferent projections to prime forebrain and hindbrain.^118^

Our group has demonstrated that TDAG8 is maximally activated by extracellular pH of 6.5 leading to intracellular increases in cAMP and pCREB *in vitro*.^134^ Presence of an acid sensor within brain areas specialized for sensing the internal milieu is important given the relevance of interoceptive sensing in PD. Ongoing studies by our group are investigating the contributions of TDAG8 to panic-relevant responses using TDAG8-deficient mice and translational rodent models of panic phenomenon.^108, 141^ Preliminary evidence supports attenuation of CO_2_-evoked fear responses in TDAG8^−/−^ mice.^108, 141^

### Chemosensory neurons in the periaqueductal gray

The recent development of rodent models of panic-like behaviors and physiology highlight the importance of the periaqueductal gray (PAG) in panic responses.^14^ Electrical and chemical stimulation of the dorsal PAG evokes panic-like responses including freezing, flight, tachycardia, tachypnea and hyperventilation^10, 142, 143^ in lower animals and stimulation of this region in neurosurgical results in similar behaviors in humans.^144^ Several structural neuroimaging studies reveal increased gray matter volumes in the midbrain and rostral pons as well as PAG, in PD patients compared with healthy controls.^145, 146^ Thus, the PAG may represent an attractive site for chemosensory pH sensing and its translation to panic expression. Focal lesions of the PAG do not alter ventilation during normocapnia, however, lead to reduced ventilatory responses to 7% CO_2._^107^ There is now strong evidence that the dorsal PAG contains chemosensitive neurons that may be intrinsically sensitive to O_2_ reduction, a hypoxia-sensitive alarm. Intravenous potassium cyanide, which produces anoxia, produced panic-like responses such as freezing and flight^147^ and when paired with 8 or 13% CO_2_ inhalation, enhances evoked flight responses and, independently facilitates the panic-like responses of rats during electrical PAG stimulation. In addition, electrical lesioning of the PAG attenuates, if not completely abolishes, panic-like responses, raising the possibility that the PAG harbors an anoxia-sensitive suffocation alarm in which dysfunction causes hypersensitivity to CO_2_. Given the extensive clinical and preclinical studies supporting a role of the PAG in behavioral and physiological panic-associated responses, presence of chemosensitive targets in this region is of interest. However, additional studies are required to determine the involvement of this proposed PAG suffocation alarm in PD.

## Translational relevance of acid-chemosensory mechanisms: from animal models to PD

Findings in translational rodent models of panic have provided information on potential target receptors and ion channels, as well as brain areas that may contribute to the pathophysiology of panic in humans (Figure 2). Clinical studies over the years have shown that a problem with acid–base homeostasis may exist in PD subjects pointing to the relevance of pH sensing and transduction targets as well as underlying circuits that contribute to pathophysiologic responses. For some acid chemosensors such as the ASIC1 channels, evidence from preclinical work translates well to human PD. Genetic studies have shown an association of polymorphisms in the ASIC-1a gene with PD.^105^ However, regional attributes and circuits underlying ASIC contributions to PD are less clear. A role of ASIC1 in the amygdala function is further supported in a rodent CO_2_ studies; however, it is noteworthy that Urbach–Wiethe subjects with damaged amygdala elicited panic and fear response to CO_2_ supporting the relevance of exrtra-amygdalar chemosensory in interoceptive threat detection and translation into panic. Here again, translational studies have suggested that dorsomedial hypothalamus, PAG, brain stem raphe and CVOs are potential sites of interest to PD. In some cases, findings from separate studies converge on selective sites that may be of particular relevance to PD. For example, studies on sodium lactate infusion in rodents by Shekhar's group highlight an important role of CVOs, such as the subfornical organ and organum vasculosum of the lamina terminalis as sites for initiation of panic responses evoked by lactate.^118^ We recently demonstrated abundance of an acid-sensing TDAG8 receptor in these areas that are recruited in panic responses to CO_2_ inhalation, a panicogen.^108, 141^ Thus, areas devoid of a blood–brain barrier that can sense central and systemic homeostatic mileau may represent upstream detection sites for panic initiation especially given their connectivity to downstream sites for expression of behavioral and physiological responses as well as the forebrain regions such as the insula that have been implicated in PD. Acid chemosensors on orexin and 5-HT neurons provides further insights into integration of interoceptive pH fluctuations leading to behavioral and respiratory arousal. Association between polymorphisms in the TPH-2 gene and susceptibility to panic symptoms evoked by CO_2_ inhalation in humans,^96^ suggests the potential convergence of serotoninergic and CO_2_ chemosensory mechanisms. Future studies focusing on the crosstalk between exteroceptive and interoceptive pathways and mechanisms will be crucial to fully appreciate the unique pathophysiology of PD.

## Potential therapeutic agents targeting pH chemosensory mechanisms

A role for pH and chemosensory mechanisms in panic physiology is increasingly appreciated on the basis of the recent explosion of data from preclinical studies; however, the translation to humans remains somewhat unclear and the degree to which these systems may be targeted by psychopharmacologic interventions is relatively unexplored. Thus, the lack of investigations related to pH or chemosensory modulating therapeutics is of high public health significance given that therapeutic options for PD are limited. As described above, selective serotonin reuptake inhibitors are the most commonly prescribed medications and, although they are effective in some patients, there are numerous side effects, delays in onset of action, whereas other modalities such as benzodiazepines—which have rapid onset of action—are associated with distinct limitations, including the risk of dependence and withdrawal. To date, there have been no clinical studies in PD patients on interventions targeted towards control of pH imbalance or chemosensory ion channel or receptor blockers. It is evident that antagonists for chemosensors such as ASIC1 ion channel may be a promising therapeutic target for PD. ASIC ion channel antagonist, such as amiloride have held promise for other acidosis associated conditions such as pain, stroke, migraine, spinal cord injury and multiple sclerosis (reviewed in Wemmie^148^). Recent studies show neuroprotective effects of amiloride in patients with progressive multiple sclerosis,^149^ supporting the safety and efficacy of this agent for alleviating pH-associated pathophysiology. Another promising target for PD are orexin receptor antagonists. Pre-treating with Orexin-1 receptor antagonist, SB334867, attenuates anxiety-like responses to CO_2_ inhalation in rats.^124^ In addition, silencing ORX precursor gene expression in the dorsal medial-perifornical hypothalamus/perifornical region, or systemically pretreating rats with SB334867 blocked intravenous sodium lactate-induced anxiety-related behavior and cardioexcitation.^119^ SB334867 also attenuated anxiety and panic-relevant behaviors induced by benzodiazepine inverse agonist FG-7142 and adenosine receptor antagonist, caffeine suggesting that orexin antagonists act on pathways common to diverse triggers of panic.^115^ In view of the relevance of acid–base dysregulation and CO_2_ sensitivity to panic it would be of interest to discuss studies on carbonic anhydrase inhibitor, acetazolamide in panic patients. Acetazolamide blocks the facilitated conversion of carbon dioxide to carbonic acid and finally bicarbonate and hydrogen ions, leading to a significant increase in CO_2_ concentration. It was hypothesized that acetazolamide would induce panic attacks in PD patients due to a rise in CO_2_ concentration. Contrary to this, panic attacks were not induced by acetazolamide.^53, 150^ In fact, it was suggested that acetazolamide may serve as an anti-panic agent, as it buffers against CO_2_-induced hydrogen ion fluctuations.^151^ As noted in that study, acetazolamide prevents the accumulation of H^+^ ions due to inhibition of carbonic anhydrase and provides support for H^+^ ions as the primary biological effector in CO_2_-evoked panic. These studies warrant further investigation and support for the therapeutic potential of acetazolamide in PD.

## Conclusions and future directions

It is clear from the clinical and preclinical findings discussed in preceding sections that interoceptive acid/base imbalance and pH chemosensory mechanisms may contribute to certain aspects of PD, particularly uncued panic attacks. Converging evidence from neuroimaging, genetics and rodent preclinical models strongly supports that underlying abnormalities in pH homeostasis and chemosensation may be an important causative factor in panicogenesis.

However, it should be noted that, while there is significant evidence and consensus on the role of pH homeostasis and impaired acid–base buffering in patients with PD, not all data support the link between pH, specifically acidosis, and panic attacks. As described herein, clinical, genetic and preclinical studies on CO_2_ inhalation strongly support this link. Although not all panicogens (for example, sodium lactate and doxapram, which may cause respiratory distress, pH shifts or compensatory acidosis as a response to alkalosis) support the notion that acidosis provokes panic. In this regard, future studies are required to further clarify these inconsistencies.

Although pH sensing may contribute to panic, systems regulating stress, arousal, fear and anxiety are also relevant, particularly in the maintenance of the disorder. It is possible that panic vulnerability occurs due to a combined deficit in the processing of internal and external threats. The late onset of PD also suggests that more than one phenomenon is required for the manifestation of the disorder. An interesting future research direction would be to study the connectivity and crosstalk between pH chemosensory mechanisms and exteroceptive threat response systems (see Figure 2). There is also a need for the development of preclinical animal models where stress and pH chemosensory threat processing and translation should be simulated as this scenario is more likely to occur in humans. These models will also be relevant for therapeutic testing of novel agents. Another important area would be to study the interaction and communication between different pH chemosensory molecules in the brain. The presence of multiple sensory mechanisms at distinct sites is reflective of a highly sensitive pH threat detection system functioning at different thresholds and sensitivities, which may be relevant to PD. In conclusion, pH homeostasis and chemosensation remains an important area of investigation that furthers our understanding of panic pathophysiology and treatment.

## Figures and Tables

**Table 1:** Studies of pH or lactate-related brain changes in patients with panic disorder

*Reference*	*Population*	*Panicogen or challenge*	*Region of interest*	*Finding*
27	Panic disorder (treated), *n*=7 Healthy subjects, *n*=7	Hyperventilation	Whole brain	↑ Brain lactate in panic disorder patients, compared with healthy subjects.
37	Panic disorder, *n*=13 Healthy subjects, *n*=10	Lactate infusion	Insula	> And longer brain lactate responses in the insula during and after lactate infusion in panic disorder patients, compared with healthy controls. Lactate responses did not normalize following fluoxetine treatment (3–4 months duration).
29	Panic disorder, *n*=15 Healthy subjects, *n*=10	Lactate infusion	Whole brain	↑ Global brain lactate increases following lactate infusion in panic disorder patients compared with healthy subjects. No lateralization of brain lactate response. No regional loci of elevated lactate observed.
26	Panic disorder (treated), *n*=9 Healthy subjects, *n*=11	Hyperventilation	Whole brain	Lower pCO_2_ during hyperventilation and slower pCO_2_ recovery following hyperventilation in patients with panic disorders compared with healthy controls.
29	Panic disorder, *n*=15 Healthy subjects, *n*=15	Visual stimuli	Occipital cortex	Greater ↑ in stimulation-related lactate/*N*-acetylaspartate in patients with panic disorder compared with healthy subjects.
36	Panic disorder, in remission, *n*=13 Panic disorder, *n*=8 Healthy subjects, *n*=12	Visual stimuli	Occipital cortex	Panic disorder patients (regardless of remission status) had greater activity-dependent ↑ in brain lactate compared with healthy subjects.
40	Healthy subjects, *n*=6	Inhalation of room air, 5% CO_2_ or hyperventilation. Visual stimuli	Whole brain	T1ρ (surrogate marker of brain pH) is a valid marker for pH changes in human brain and changes seen are dependent on brain activity.
41	Panic disorder, *n*=13 Healthy subjects, *n*=13	Visual stimuli	Occipital cortex	↑ Activity-dependent T1ρ in panic disorder patients compared with healthy subjects.
39	Panic disorder, *n*=15 Healthy subjects, *n*=12 Experienced divers, *n*=15	7% CO_2_ inhalation	Brainstem	↑ Brain stem activation in patients with panic disorder in response to CO_2_ inhalation as compared with both healthy controls and experienced divers.

## Notes

JRS receives or has received research support from Edgemont, Eli Lilly, Shire, Forest Research Laboratories, Lundbeck, the American Academy of Child and Adolescent Psychiatry and the National Institute of Mental Health, and is an employee of the University of Cincinnati. He receives royalties from Springer Publishing for two books. RS is a consultant for Ono Pharmaceuticals, Japan. The remaining author declares no conflict of interest.
